# Novel Bacterial Cellulose/Gelatin Hydrogels as 3D Scaffolds for Tumor Cell Culture

**DOI:** 10.3390/polym10060581

**Published:** 2018-05-24

**Authors:** Jing Wang, Li Zhao, Aixia Zhang, Yuan Huang, Javad Tavakoli, Youhong Tang

**Affiliations:** 1 Key Laboratory of Advanced Textile Composite Materials of Ministry of Education, Institute of Textile Composite, School of Textile, Tianjin Polytechnic University, Tianjin 300387, China; 2 Institute for Nano Scale Science and Technology, Medical Device Research Institute, Flinders University, Bedford Park, SA 5042, Australia; javad.tavakoli@flinders.edu.au; 3 Pathology Department, The First People’s Hospital of Xuzhou, Xuzhou 221002, China; zhaolityzl@aliyun.com; 4 Medical Oncology, Affiliated Hospital of Shandong Academy of Medical Sciences, Jinan 250031, China; doctorzhangax@163.com; 5 School of Materials Science and Engineering, Tianjin University, Tianjin 300072, China; yi_huangyuan@tju.edu.cn

**Keywords:** bacterial cellulose, gelatin, 3D scaffolds, biocompatible, hydrogel

## Abstract

Three-dimensional (3D) cells in vitro culture are becoming increasingly popular in cancer research because some important signals are lost when cells are cultured in a two-dimensional (2D) substrate. In this work, bacterial cellulose (BC)/gelatin hydrogels were successfully synthesized and were investigated as scaffolds for cancer cells in vitro culture to simulate tumor microenvironment. Their properties and ability to support normal growth of cancer cells were evaluated. In particular, the human breast cancer cell line (MDA-MD-231) was seeded into BC/gelatin scaffolds to investigate their potential in 3D cell in vitro culture. MTT proliferation assay, scanning electron microscopy, hematoxylin and eosin staining and immunofluorescence were used to determine cell proliferation, morphology, adhesion, infiltration, and receptor expression. The in vitro MDA-MD-231 cell culture results demonstrated that cells cultured on the BC/gelatin scaffolds had significant adhesion, proliferation, ingrowth and differentiation. More importantly, MDA-MD-231 cells cultured in BC/gelatin scaffolds retained triple-negative receptor expression, demonstrating that BC/gelatin scaffolds could be used as ideal in vitro culture scaffolds for tumor cells.

## 1. Introduction

There are over 200 different types of cancer in the world. It is predicted that one in three people will develop some form of cancer in their lifetime. Although mortality rates from cancer have been decreasing over the past three decades, it still accounts for one in four deaths. At the same time, many effective treatments and drugs have been developed, but they do not always stop the spread or recurrence of the disease [1]. In order to predict the clinical efficacy of cancer treatments clearly, many researchers focus on cancer cell growth, apoptosis, and resistance to drugs via in vitro cell culture models or animal models. Yet current preclinical research suffers from critical drawbacks, and examination of therapeutic efficacy using cancer cell lines in many cases, such as plate cultivation, does not accurately predict clinical effectiveness. Testing in animal models tends to be financially demanding and takes a long time [2]. To solve such problems, the establishment of novel systems will be necessary for better assessment of anti-cancer agents. In vitro tumor models are invaluable systems for studying the dynamic and progressive behavior of cancer under controlled conditions [3,4]. Hence, there is a need for the in vitro tumor model that would mimic the 3D structure and microenvironment of tumors.

3D assays are known to simulate in vivo cellular conditions better than traditional 2D plate cultivation systems. 3D assays influence the formation of a subpopulation of cancer cells with stem-cell-like properties, providing new insights into cancer treatment and cancer stem cell research. Therefore, 3D cell culture might bridge the gap between animal models and universal human studies [5]. Compared with 2D cell culture, 3D cell culture provides a large area for cell attachment and growth [6]. Many important signals, tissue phenotypes and key regulators can be preserved in 3D cell culture [7]. Hence, if cancer cells can be cultivated without losing their original traits under culture conditions, it is highly likely that those culture conditions are suitable for experimental platforms to evaluate therapeutic efficacy. Cancer cells via in vitro 3D culture are able to overexpress pro-angiogenic growth factors and “stemness” genes [8,9]. It was recently demonstrated that in vitro 3D culture models show a more realistic drug response, thus supporting improved drug-resistance studies.

Recently, scientists have begun modifying culture components to better mimic in vivo conditions. Various polymers have been investigated as scaffolds for 3D culture. Sahoo’s group prepared poly(lactic acid) (PLA) and poly(lactic-*co*-glycolide) (PLGA) scaffolds with poly(vinyl alcohol) (PVA) via a solvent-evaporation method for 3D cell culture. Although the scaffolds supported cell adhesion and cell growth better than other formulations of micro particles after 7 days culture, the samples for fabricating the scaffolds were low in yield and small in size, which was not beneficial for 3D cell culture and detection. The growth of cell was characterized via cell proliferation and microscope photographs without other biological tests. Hence, the growth of cells within micro particles is not known [10]. Talukdar and colleagues obtained silk fibroin protein for an in vitro tumor model. Cells cultured on the scaffolds obtained from silk fibroin protein could be viably maintained for long-term culture, e.g., 8 weeks. However, silk fibroin protein is expensive and unstable, limiting its wide application [5]. In a study by Szot and colleagues [11], electrospun polycaprolactone (PCL)/collagen I scaffold was investigated as a potential 3D scaffold for an in vitro cancer model. The results showed that cells could spread well after 7 days culture. However, the electrospun fibers did not allow proper infiltration of the cells to the core of the scaffolds, due to the limited pore size and thickness. The cells could not be found inside the scaffolds. Other important disadvantages of electrospun fibers are that the fiber diameters are usually at the upper limits of the 50–500 nm range, which is different from the range of extracellular matrix (ECM), and the specimen thickness is limited.

These requirements and the tremendous interest have stimulated researchers to develop more materials and techniques for 3D culture. Among the natural polymers, bacterial cellulose (BC) has been shown to be interesting as a biomaterial. It is synthesized extracellularly by the bacterium *Acetobacter xylinum (A.xylinum X-2)* and possesses some unique mechanical, physical and biological properties [12,13,14,15]. Due to its excellent properties, including remarkable mechanical properties in both dry and wet states, high moldability, high water-holding capacity and high porosity, BC has been investigated as scaffolding in tissue engineering studies, with results demonstrating suitable cell proliferation, differentiation and adhesion [16,17,18]. Although it shows superiority to other scaffolds, BC has some disadvantages. As a kind of nonvalent polysaccharide, BC in its natural state has no micro pores (>100 μm), no influence on the concentration of elastase in vitro, little antioxidant capacity, and little biological activity. Some methods of improving pore size or porosity of BC have been reported. Hu et al. used a combination method consisting of acetic acid treatment and freeze-drying operation to improve the porous profile of BC. This technology was a simple and fast method which could improve in the porosity of the inner structure of BC [19]. In other study, microporous BC scaffolds were prepared by incorporating 300–500 μm paraffin wax microspheres or starch particles into the fermentation process. After harvest BC, paraffin wax microspheres and starch particles were removed by NaOH solution [18,20]. These BC scaffolds with micropores were used for human fibroblast cells, smooth muscle cells (SMCs), and MC3T3-E1 osteoprogenitor cells culture in vitro. Xiong’s group [21,22] employed porous BC for cancer cell culture in vitro. Although BC showed no negative effects on cell viability and proliferation, cancer cells could spread on BC scaffolds. However, cells could not form multilayers and clusters until 28 days culture. After 7 days culture, very few cells were found inside scaffolds close to the inoculated cell area. Therefore, some molecules with excellent bioactivity were introduced into BC networks, including collagen, chitosan, hydroxyapatite, bone morphogenetic protein and so on. As a derivative of collagen and a polypeptide derived from an extracellular matrix, gelatin is biodegradable, inexpensive, has good biocompatibility, low immunogenicity, desirable adhesiveness, promotion of cell adhesion and growth. Wide applications of gelatin-based scaffolds have been demonstrated in different areas of tissue engineering [23,24]. We previously prepared and characterized BC/gelatin hydrogels via crosslinking. In that study, the experimental parameters for preparing BC/gelatin hydrogels were clarified. The hydrogels retained adequate network and biocompatibility [25]. However, the potential and biological activity of BC/gelatin for tissue engineering applications and in vitro 3D culture have not yet been studied systematically.

In this study, a stable and malignant triple-negative breast cancer (TNBC) cell line called human breast cancer cell line (MDA-MD-231) was chosen to seed onto BC/gelatin hydrogels to analyze cell behavior such as viability, proliferation, adhesion and morphology. Investigation of the cancer cellular responses to the scaffolds to evaluate BC/gelatin used cancer cell in vitro 3D culture.

## 2. Experiment, Materials, and Methods

### 2.1. Materials

Materials used: *A.xylinum X-2*; glucose, peptone, yeast extract, disodium phosphate, acetate acid, sodium hydroxide, ethyl alcohol, formaldehyde, glutaraldehyde, sodium chloride and agar (Sigma, Shanghai, China); deionized water; Hanks’ Balanced Salt solution, Notch 1 Antibody (Notch 1), Diaminobenzidine (DAB) Substrate Kit, phosphate buffered saline (PBS) and dimethyl sulfoxide (DMSO, Gibco, Shanghai, China); gelatin (Sigma, Analytical grade, Shanghai, China). All agents were used as received without further purification.

### 2.2. Preparation of BC and BC/Gelatin Scaffolds

Preparation of BC: the *A.xylinum X-2* was grown for 5~7 days in a static culture (2.5% glucose, 0.75% peptone, 1% yeast extract and 1% disodium phosphate (*v*/*v*)). The initial pH value was adjusted to 4.0~5.0 by acetate acid. BC fabric was harvested and purified by boiling in deionized water several times and boiled in a 0.5 M NaOH solution for 30 min, and then rinsed several times with deionized water until a neutral pH was obtained. The BC fabrics were then stored in deionized water at room temperature prior to use.

Preparation of BC/gelatin hydrogels: BC/gelatin hydrogels were prepared via procyanidin (PA) crosslinking, which was a useful method to prepare the hydrogel for tissue engineering. BC films were immersed in an aqueous gelatin solution (0.25 wt %). The solution with BC was kept at 37 °C in a shaking incubator at 160 rpm rotational speed for 24 h. Then the soaked BC films were placed in PA solution (95%, 0.05 wt %) to finish crosslinking. The crosslinking was completed in the same incubator at 180 rpm for 2 h. The resultant BC/gelatin hydrogels were used in further analyses. The scaffolds before use were frozen at −70 °C for 6 h, and then freeze-dried at −50 °C for 12 h.

### 2.3. Characterization of Materials

FE-SEM: the surface structures of samples were observed by field emission scanning electron microscopy (FE-SEM, Nanosem430 microscope, FEI, OR, USA). For FE-SEM observations, samples were sputter coated with gold and were observed at an accelerating voltage of 10 kV. The average fiber diameter of nanofibers was determined by measuring the diameters of the nanofibers at 100 different points on an FE-SEM image. The diameters were presented as the average ± standard deviation (SD).

Mercury intrusion porosimeter: the porosity and surface area of the BC/gelatin hydrogels were determined by a mercury intrusion porosimeter (PoreMaster 60 GT, Quantachrome Instruments, FL, USA) which could measure pore size ranging from 3.6 nm to 950 μm.

FTIR analysis: Fourier transform infrared spectroscopy (FTIR, Nicolet Magna IR-600, Nicolet, WI, USA) was performed to characterize samples at the room temperature. All spectra were recorded by transmittance mode (32 times scanning, 400–4000 cm^−1^).

Water contact angle measurement: samples were measured using a contact angle instrument (dataphysics OCA15EC, dataphysics, Filderstadt, Germany) at room temperature. All measurements were determined by averaging sample values at five different points.

Mechanical testing: a testing system (CSS-44100) with a 250 N load cell was used to test the mechanical properties of samples. Samples were measured at the speed of 5 mm/min, and the modulus was determined. The mechanical properties of all samples in the wet state were determined in accordance with ASTM D 638-98 (Lab Sans, Shenzhen, China). The average values were calculated from 5 samples per group.

### 2.4. Cell Study

The cells were cultured in high-glucose Dulbecco’s modified Eagle’s medium (Gibco, Shanghai, China) supplemented with 10% fetal bovine serum (FBS, Gibco, Shanghai, China). The cells were then trypsinized and seeded onto scaffolds using the density of 2 × 10^5^–5 × 10^5^ cells /scaffold. The cells on the scaffolds were allowed to grow for 0–7 days for different analyses under standard conditions (37 °C, 5% CO_2_).

MTT proliferation assay: cells (2 × 10^5^–5 × 10^5^) were seeded onto the scaffolds (diameter: 10 mm, height: 1 mm) under the aforementioned condition. After 1, 3, 5 and 7 days culture, the cell-scaffold constructions were rinsed with PBS to remove non-adhering cells, followed by incubation in 50 μL 3-(4,5-Dimethylthiazol-2-yl)-2,5-diphenyltetrazolium bromide (MTT) reagent for 4 h under the same conditions described. After removal of the medium, the converted dye was dissolved in DMSO (500 μL /well). Solution (150 μL) from each sample was transferred to a 96-well plate. Absorbance of the converted dye was measured at the wavelength of 490 nm. Proliferation measurement data were collected from triplicate samples and expressed as the mean ± SD.

Cell imaging: cell adhesion and images were determined using SEM (JEOL JSM-6360LV). After 3 days culture, the cell-scaffold samples were fixed using 4% glutaraldehyde for 12 h, dehydrated in increasing concentrations of alcohol, i.e., 40%, 50%, 60%, 70%, 80%, 90% and 100%, air-dried or freeze-dried, and sputter coated with gold and observed at an accelerating voltage of 10 kV. The freeze-dried samples used for surface and cross-section observation were fixed on the sample table without other treatment.

Hematoxylin and eosin (H&E) staining: the cell-scaffold constructions were washed with ice-cold normal saline (0.9% NaCl), cut transversely into thin slices (5 μm), and then fixed into 10% neutral-buffered formaldehyde for 24 h. The tissues were then transferred into 70% ethyl alcohol, processed, and embedded in paraffin wax. The sections with cells were stained with H&E staining for histological examination under a light microscope.

Immunofluorescence: to analyze the cells by fluorescence microscopy and detect differences in cell growth on BC/gelatin scaffolds, immunofluorescence was used. Cells cultured in BC/gelatin scaffolds were fixed in 10% buffered formalin for 1 day, dehydrated in 50%, 70%, 95% and 100% ethanol for 15 min each time, and then submerged in xylene twice for 10 min each time. Paraffin-embedded tissue blocks were prepared and cut with a microtome. Put the blocks into antigen retrieval, prepared using 0.01 M citrate buffer (pH 6.0), in a microwave oven at high power up to boiling for 3 min, then low at power for 10 min to maintain the temperature, followed by cooling down at room temperature for at least 30 min, and rinsing twice with distilled water. Pre-incubated sections were treated with 3% H_2_O_2_ for 10 min, then rinsed three times with PBS for 10 min each time, encapsulated with 10% goat serum and maintain them at room temperature for 10 min and rinsed with deionized water. Sections were incubated at 4 °C with Notch 1 (dilution ratio 1:200) overnight, then incubated for 1 h at room temperature to recovery temperature, and rinsed three times with PBS, for 10 min each time. Subsequently, sections were incubated at 37 °C with biological enzyme for 20 min, then rinsed three times with PBS for 10 min each time. Sections were incubated at 37 °C with horseradish peroxidase-labelled streptomycin for 20 min. Pre-incubated sections were stained with DAB for 3–10 min and subsequently stained with hematoxylin for 5–10 min and washed several times with tap water. 1% HCl solution (acid alcohol solution 1%, hydrochloric acid, 1 mL 70% ethanol) and ammonia water (ammonia water solution, 0.2%) were used for differentiation. Sections were washed with tap water several times. Subsequently, they were rinsed with 100% ethanol twice and 5 min each time; 90% ethanol for 5 min; 70% ethanol for 5 min. xylene twice and 5 min each time and rinsed in distilled water. Sections were dried overnight in the oven or dehydrate with ascending ethanol. Mount with coverslips using mounting medium. Finally, sections on glass slides were observed under a microscope.

### 2.5. Statistical Analysis

All data are expressed as means ± SD. One-way analysis of variance was used to analyze statistical differences among multiple comparisons. A value of *p* < 0.05 was considered to be statistically significant.

## 3. Results and Discussion

### 3.1. Morphology of BC and BC/Gelatin Hydrogel

**Figure 1:**
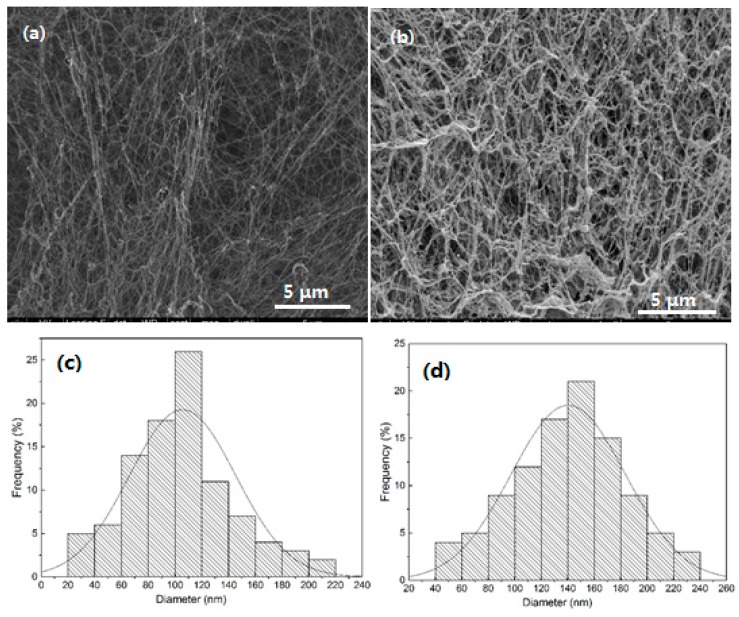
(**a**,**b**) SEM images and (**c**,**d**) diameter distributions of (**a**,**c**) pure BC and (**b**,**d**) BC/gelatin hydrogel.

Figure 1 shows FE-SEM images and diameter distribution of the pure BC and BC/gelatin hydrogel. 3D network structures made up of a random assembly of fibrils are observed. The pure BC shows interconnecting pores that conform to the cellulose structure of tissue engineering. It is noted that the pore size varies within a range of tens to hundreds of nanometres. After gelatin was introduced, the network is preserved, as shown in Figure 1b. Figure 1b also shows that thin gelatin coatings have formed and are wrapped around the nanofiber surfaces. Note that, after combining with other materials, each BC nanofiber is uniformly wrapped by other crystals and the interconnecting spaces are still evident, a feature which is important for tissue engineering scaffolds. The average diameter of pure BC nanofibers was determined to be 100 ± 20 nm, as shown in Figure 1c, and the average diameter of BC/gelatin fibres was 143 ± 20 nm, as shown in Figure 1d. Hence, the average thickness of the gelatin coatings was about 20 nm.

Scaffolds must meet certain physical and biological properties for tissue engineering applications. An ideal scaffold should possess 3D connected porous structure to facilitate cell migration, proliferation, and differentiation. Table 1 presents the porosity and surface area of the pure BC and the BC/gelatin hydrogel. According to the results, the measured porosity and specific surface area of the BC/gelatin scaffold, 86.05% and 84.30 m^2^/g respectively, were comparable to those of the pure BC scaffold at 92.05% and 91.50 m^2^/g respectively. Although the formation of the thin gelatin coatings decreased the porosity BC/gelatin hydrogel. BC/gelatin hydrogel still possessed a suitable structure for tissue scaffold materials.

### 3.2. Chemical and Surface Structure of BC and BC/Gelatin Hydrogel

**Figure 2:**
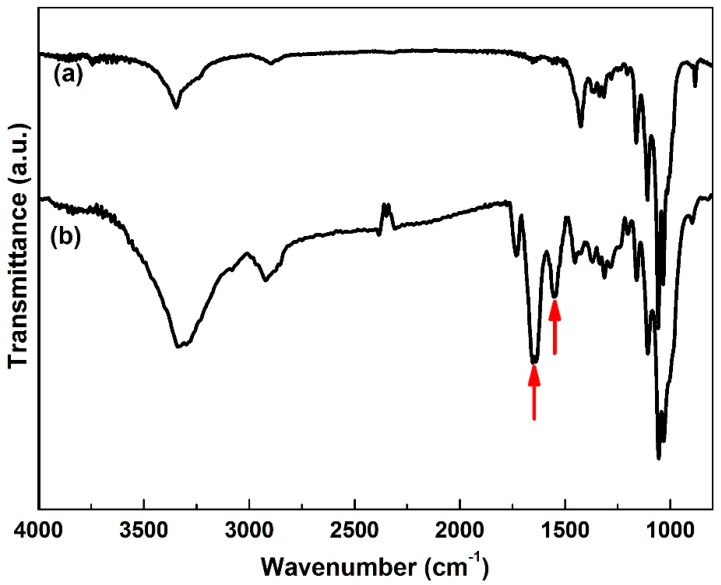
FTIR spectra of (**a**) pure BC and (**b**) BC/gelatin hydrogel.

Figure 2 shows FTIR spectra of pure BC and BC/gelatin samples. The characteristic bands of pure BC are nearly identical to those reported in other research [12].The characteristic absorption bands are observed in both pure BC and BC/gelatin samples. Meanwhile the amide I and amide II bands of gelatin (indicated by arrows) at 1640 and 1540 cm^−1^ are observed in the spectrum of the BC/gelatin [25,26,27]. In addition, in our previous studies, amide I band showed red-shifted which proved that some hydrogen bonds are formed in the order region of the amide I band [25]. In Figure 2b amide I band (a characteristic frequency of 1670 cm^−1^) displays red-shifted too (from 1670 to 1640 cm^−1^).This result proves that gelatin is incorporated into the BC network after the crosslinking process, coinciding with the above FE-SEM results.

**Figure 3:**
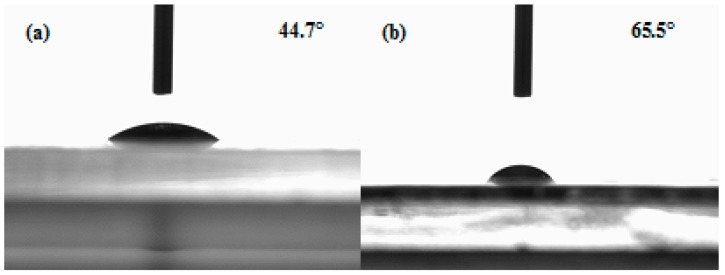
Water contact angle of (**a**) BC and (**b**) BC/gelatin hydrogel.

Figure 3 shows the water contact angle of the pure BC and BC/gelatin hydrogel, showing that the pure BC and the BC/gelatin hydrogel are hydrophilic. Their water contact angles are 44.7° ± 1.9° and 65.5° ± 2.3° respectively. The increased water contact angle suggests that the introduction of gelatin significantly decreased the surface hydrophilicity due to the change in surface composition. The presence of gelatin coating on the fiber surface reduces the amount of free hydroxyl radical on fiber surface and affects the hydrophilicity of substrate. Compared with the pure BC, the water contact angle of the BC/gelatin increased and the hydrophilicity was weakened. However, compared with other scaffolds, the BC/gelatin hydrogel possessed good hydrophilicity and could facilitate nutrient solution into the inner part of the scaffold, which is important for tissue engineering and in vitro tissue culture [28].

### 3.3. Mechanical Properties of BC and BC/Gelatin Samples

Table 2 shows the tensile strength, Young’s modulus, and elongation at break of the pure BC and the BC/gelatin hydrogel. Clearly, the two samples exhibit similar mechanical properties in strength and modulus. The BC/gelatin hydrogel has slightly lower tensile strength, Young’s modulus and elongation at break than the pure BC. This is because the mechanical properties of gelatin are lower than those of BC. After the formation of thin gelatin coatings on the nanofiber surface, the polyhydroxy structure of cellulose is affected, and the hydrogen bonding between fibers is destroyed. Hence, the mechanical properties decrease slightly. However, compared with previous reports of scaffold materials [29], the BC/gelatin hydrogel showed superior mechanical properties, suggesting that modified BC/gelatin hydrogels might be suitable for scaffold materials.

### 3.4. Cell Viability, Proliferation and Morphology in Scaffolds

**Figure 4:**
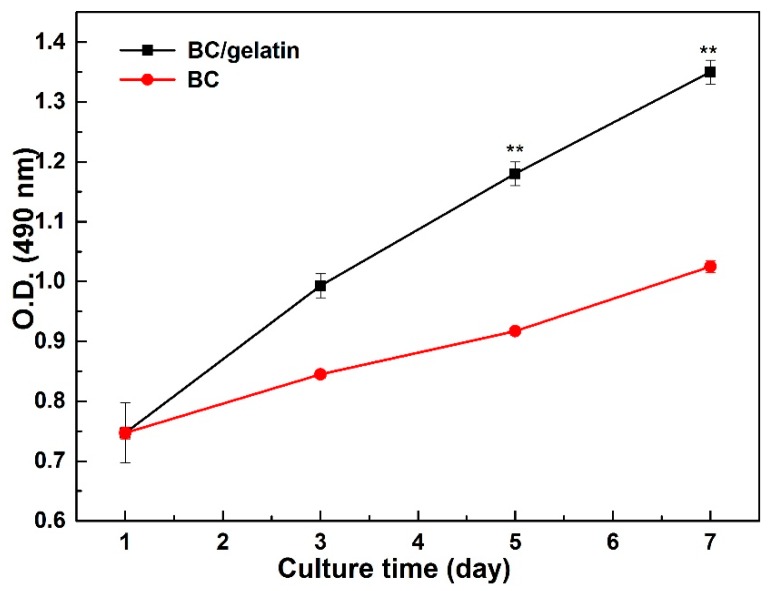
The proliferation of MDA-MB-231 cells seeded in pure BC and BC/gelatin scaffolds for 7 days. ** *p* < 0.05.

In this study, BC/gelatin hydrogels were used for 3D culture of tumor cells to evaluate the potential for imitating the tumor microenvironment in vitro. First, MTT assay was carried out to test the cytotoxicity of the BC/gelatin hydrogel. MDA-MB-231 cells were seeded into pure BC and BC/gelatin hydrogel. Figure 4 shows the MTT assay results. During 7 days culture, the cells are viable and their proliferation is robust, maintaining a constant rate in both scaffolds. When both scaffolds were compared, the cell viability and proliferation of the BC/gelatin hydrogel were better than those of the pure BC. The difference in cell viability and proliferation proved that the content of bioactive molecules was crucial to the in vitro culture of tumor cells. Furthermore, tumor cells showed stronger vitality and proliferation ability than normal tissue cells [30,31]. In this result, cell proliferation on BC/gelatin after 3 days is similar to that on BC scaffolds after 7 days. Aging is a very important factor in the detection of tumor in vitro. In the previous findings although the tumor cells grew well in BC scaffolds with macropores after 28 days culture, the time required was too long and affected clinical and research applicability [21]. In porous BC scaffolds, very few cells were distributed within the scaffolds after 7 days culture, making it difficult to detect cells [22]. Here, the contrasting experimental results showed that cancer cells seeded into BC/gelatin hydrogel could effectively shorten the biological detection time. These preliminary findings suggested that BC/gelatin hydrogel scaffolds were more suitable for in vitro 3D culture of tumor cells than pure BC scaffolds, and could be used for cancer cell in vitro culture.

To confirm cell morphology on the surface and inside of scaffolds, cell adhesion and spreading, as well as cell interaction with the nanofibers of scaffolds, were evaluated using SEM. A previous study [11] demonstrated that, compared with collagen scaffolds, MDA-MB-231 cells grown on pure BC scaffolds did not spread out on the surface or inside of scaffolds. Other studies proved that BC scaffolds with manufactured porosity could support cell growth and adhesion. Cell aggregates attached and spread throughout the surface of the scaffolds and connected to neighboring cells [19,20]. However, cells had to be incubated for 28 days before they began to attach and spread, a finding consistent with our MTT experimental results. Through cultivation of 28 days or longer, the cells grew well, a result similar to ours, but that culture duration was too long. After long-term cell cultivation, mutation in reproductive cells and the expression of receptor of specific cancer cells cannot be determined, data which are important for subsequent clinical treatment.

**Figure 5:**
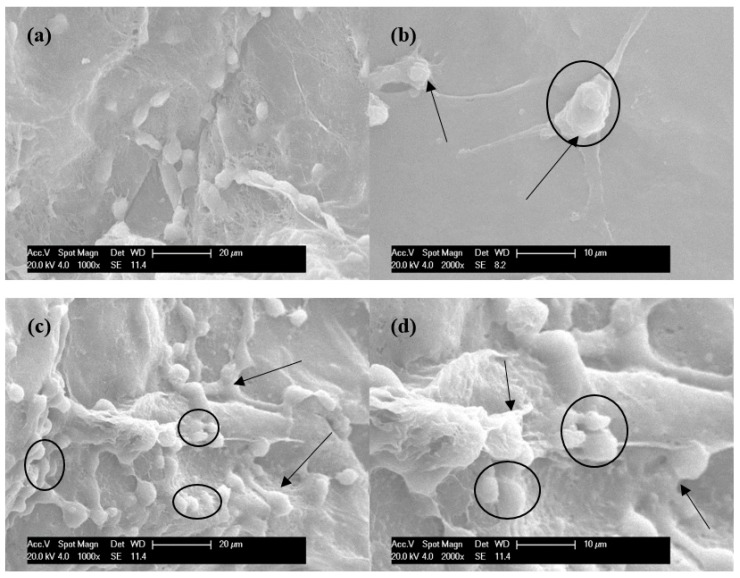
Morphology of MDA-MB-231 cells seeded on the surface of (**a**,**b**) pure BC and (**c**,**d**) BC/gelatin scaffolds after 3 days culture. The arrows and ellipses indicate cells adhering to BC scaffolds by discrete filopodia.

In our in vitro studies, the adhesion, viability and morphology of cancer cells after 3 days culture on pure BC and BC/gelatin hydrogels were observed. Figure 5 shows the SEM images of MDA-MB-231 cells. As the images show, cells adhered to both the surface and the inside of pure BC (Figure 5a,b) and BC/gelatin (Figure 5c,d) scaffolds by discrete filopodia (shown by arrows). Comparison of cells grown on pure BC and those grown on BC/gelatin indicates that, even after 3 days, the latter display a higher number of adhesive structures than cells grown on pure BC scaffolds. It is interesting to see that cells which exhibited their characteristic morphology of a roughly rounded shape tended to attach to and grow along the scaffolds (shown within the ellipses). This phenomenon was more evident in the BC/gelatin scaffolds than in the pure BC scaffolds. This cell growth on the BC/gelatin scaffold was similar to that found in cells seeded into chitosan-alginate scaffolds [32]. The cells were transformed into round shapes with many pseudopodia bonded to the scaffolds. Note that the cells cross each other in a random and multilayered fashion, and formed clusters of cells are observed on BC/gelatin scaffolds (within the ellipse of Figure 5c,d). In vitro culture, due to the micro-structure and component limitations of cultured scaffolds, multilayered and formed clusters cells are hard to find during the short time culture [11], but in this study, cells could spread and form multilayer and clusters after 3 days culture (within the ellipses in Figure 5c,d). These results suggest that the BC/gelatin scaffolds had the potential to support the adhesion, viability and morphology of the MDA-MB-231 cells. Seeding cells into the BC/gelatin scaffold could not only reduce the amount of time needed to complete experiments but could also reduce the significant costs and loss of animal life associated with in vivo models.

**Figure 6:**
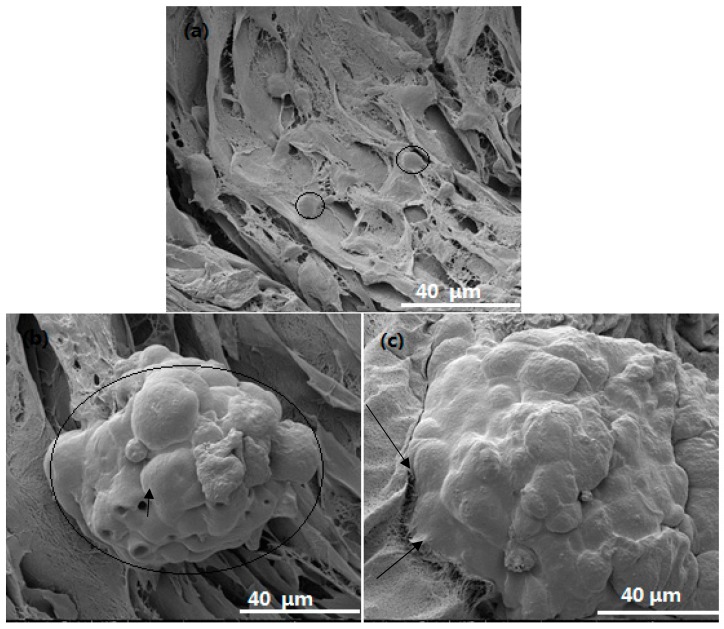
The inside of scaffolds morphology of MDA-MB-231 cells seeded within (**a**) pure BC scaffolds and (**b**,**c**) BC/gelatin scaffolds after 3 days culture. The arrows and ellipses in the images indicate cells within the scaffolds both independent and spread with pseudopodia.

FE-SEM images of cells within the pure BC and BC/gelatin scaffold after 3 days are shown in Figure 6. Initially, the cells were seeded inside the scaffold by an injection syringe. The number of cells inside the scaffolds was expected to be more than that on the surface. As shown in Figure 6, after 3 days culture, the cells within the pure BC scaffold are independent and cannot form cell clusters similar to cells growth on the surface (within the ellipses in Figure 6a). In contrast, within the BC/gelatin scaffolds, cells are aggregated into clusters with higher cell density (within the ellipse of Figure 6b). Moreover, the cells at the bottom are linked closely to the microfibers of scaffolds (the arrows in Figure 6c). Apart from cell proliferation, inert surfaces, such as catheters which are useful for nutrient transportation, are also formed between cells (the arrow in Figure 6b). Compared with the reported scaffolds within in vitro culture, all these morphological results show that BC/gelatin scaffolds have a considerable effect on cell adhesion and spreading, strongly supporting cell adhesion, colonization, spreading, proliferation and differentiation.

### 3.6. Hematoxylin-Eosin (H&E) Staining of Cells in Scaffolds

**Figure 7:**
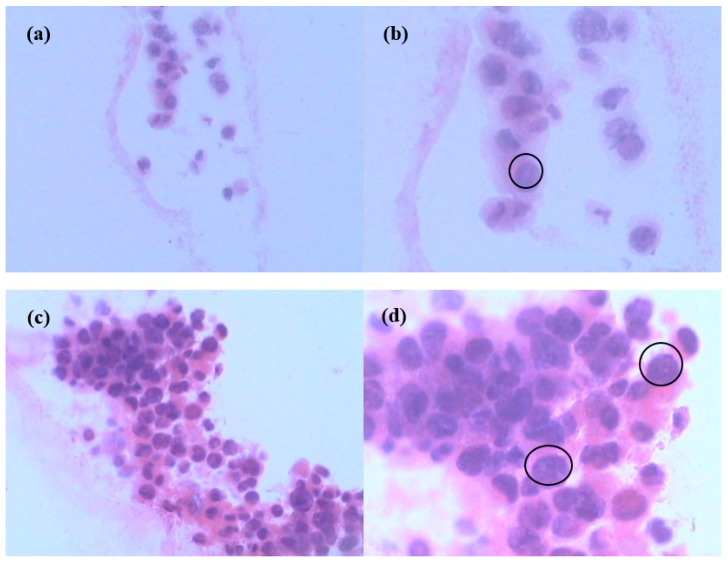
Histological analysis of cells seeded in (**a**,**b**) pure BC and (**c**,**d**) BC/gelatin scaffold for 3 days ((**a**,**c**), ×200; (**b**,**d**), ×400).

To obtain more information about cells within scaffolds, histological evaluation was performed, providing qualitative detail to detect possible changes in cell morphology and ECM production. Figure 7 shows light microscopy images of pure BC and BC/gelatin scaffolds with cancer cells with H&E staining (a single cell occupies the ellipse in Figure 7b,d). Some researchers have reported that pristine pure BC could not support cancer cell ingrowth [11]. However, other groups have reported that cells within scaffolds could be observed only in porous pure BC scaffolds with macropores. Those results proved that cancer cells migrated inside scaffolds and formed clusters in large quantities after long culture duration, such as up to 28 days [21]. In that study, a small number of cells had distributed independently after 3 days culture, not only within scaffolds but also transported to the surface of scaffolds, a finding similar to the results for pure BC, as shown in Figure 7a,b. However, sections with cells were not easily detected under light microscopy, unlike the BC/gelatin scaffolds with cancer cells (Figure 7c,d). Our results show that cells distribute well throughout the BC/gelatin scaffolds. Comparison of pure BC (Figure 7b) and BC/gelatin scaffolds (Figure 7d) after 3 days culture shows that MDA-MB-231 cells spread and infiltrated within scaffolds and many cell clusters with ECM were observed within the BC/gelatin scaffolds. Our results indicated that cells in BC/gelatin scaffold grew more successfully than those in pure BC. At the same time, cell density, number of cell clusters and ECM production show a marked increase inside scaffolds with BC/gelatin hydrogels. MDA-MB-231 cells presented ingrowth, adhesion, robust proliferation and differentiation inside BC/gelatin scaffolds after short duration culture. These results suggested that modification of BC with gelatin could be conducive to proliferation, adhesion, differentiation and ingrowth of cells. The findings indicate that BC/gelatin hydrogel could be an ideal material for in vitro 3D culture of tumor cells.

### 3.7. Immunohistochemistry (IHC) of Cells in Scaffold

Breast cancer is the main cause of death by cancer among women. A woman has a 12.3% risk of being diagnosed with breast cancer during her lifetime [33]. Breast cancer also has the highest incidence of malignant neoplasia among women. Breast cancer represents a heterogeneous cancer group, with complex biological behavior and great clinical variability [34]. Triple-negative breast cancer (TNBC) is one subgroup of breast cancer. Approximately 10–17% of all cases of breast cancer are TNBCs [35]. Although the incidence of TNBCs is less than that of other subtypes of breast cancer, its clinical prognosis is extremely poor, and there is no effective treatment. This subgroup is regarded as important clinically because of its aggressive clinical behavior, poorer patient prognosis and lack of an established therapeutic target. More important, compared to patients with other subtypes of breast cancer, TNBC patients are typically younger (<50 years) [36]. To date, not a single targeted therapy has been approved for TNBCs treatment, and cytotoxic chemotherapy remains the standard systemic treatment. Thus, TNBC is an aggressive subtype of breast cancer with a poor prognosis, and it is an important research topic among the many subgroups of breast cancer [37].

**Figure 8:**
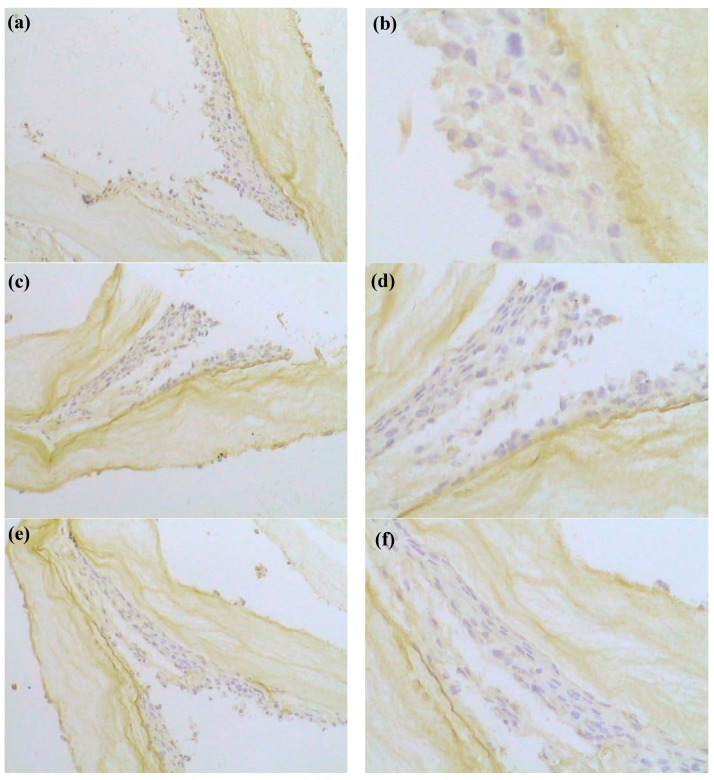
Immunohistochemical localization of (**a**,**b**) ER, (**c**,**d**) PR and (**e**,**f**) HER-2 proteins in MDA-MB-231-BC/gelatin scaffolds exposed to basal medium before and after cryopreservation. ((**a**,**c**,**e**), ×200; (**b**,**d**,**f**), ×400).

TNBCs have been defined as a subgroup with a negative expression for all estrogen receptor (ER), progesterone receptor (PR), and human epidermal growth factor receptor-2 (HER-2) [35]. In 2007, at the St. Gallen consensus meeting for decision-making about adjuvant therapies (chemotherapy, endocrine therapy and trastuzumab), operable primary breast cancers were recommended to be categorized based on the status of ER, PR and HER-2 [38]. For identification of TNBCs, the threshold of positivity for ER, PR and HER-2 may differ among cultures. Hence, characterization of the status and expression of ER, PR and HER-2 is particularly important for tumor cells cultured in vitro. In research reports of TNBCs, expression of the receptor is an indispensable test standard. Whether the receptor expression is influenced via in vitro culture is the key determinant for clinical testing [39]. In vitro culture is meaningful only if the receptor expression is not affected. However, in some previous reports of MDA-MB-231 cell lines cultured in vitro [5,21,40], characterization of the status and expression of the receptor was missing. In research reports of tumor tissue, a number of immunohistochemical studies of the TNBCs have been performed [38,39,41]. In the present study, harvested MDA-MB-231 cell lines with BC/gelatin scaffolds was immune histochemically stained for ER, PR and HER-2 expression. Figure 8 shows the IHC for ER, PR and HER-2. It shows that MDA-MB-231 cell lines are ER negative (ER (−)), PR negative (PR (−)) and HER-2 negative (HER-2 (−)), demonstrating that MDA-MB-231 cell lines cultured in vitro within BC/gelatin scaffolds retained the triple-negative expression pattern of key breast cancer markers. It is suggested, therefore, that the results of MDA-MB-231 cells cultured in BC/gelatin scaffolds could be a valuable reference for researching the biological behavior of tumor tissue, drug selection for cancer patients, clinical detection and so on. These results are important for subsequent drug testing and cancer patient treatment.

## 4. Conclusions

Bacterial cellulose (BC)/gelatin hydrogels were successfully obtained. Through the crosslink reaction, gelatin was introduced into BC scaffolds and wrapped the pure BC nanofibers. The morphology, chemical structure, mechanical properties, porosity, and wettability of the hydrogel were characterized. The BC/gelatin hydrogels were used for in vitro culture of cancer cells. A human breast cancer cell line (MDA-MD-231) belonging to a triple-negative breast cancer was seeded into pure BC and BC/gelatin scaffolds to evaluate the feasibility of scaffolds for 3D in vitro culture. The results showed that MDA-MD-231 cells with normal morphology could grow, proliferate, attach, differentiate, and penetrate into scaffolds. Moreover, such cells in BC/gelatin scaffolds showed superior vitality and formed multilayered growth and more cell clusters. For the same culture duration, cells seeded in BC/gelatin were significantly stronger than those in pure BC. Furthermore, the results of immunohistochemistry revealed clearly that MDA-MB-231 cell lines were estrogen receptor negative (ER (−)), progesterone receptor negative (PR (−)), and human epidermal growth factor receptor-2 negative (HER-2 (−)), retaining the triple-negative expression pattern of key breast cancer markers. These findings indicate that BC/gelatin scaffolds could support cell growth, promote cell proliferation, adhesion and differentiation, and possess excellent biological compatibility. Thus, BC/gelatin hydrogels would be a feasible and inexpensive candidate for tumor cells cultured in vitro for cancer biology studies, clinical diagnosis and tumor tissue engineering applications.

## Figures and Tables

**Table 1:** Porosity and surface area of BC and BC/gelatin hydrogel.

Samples	Porosity (%)	Surface Area (m^2^/g)
BC	92.1	91.5
BC/gelatin	86.1	84.3

**Table 2:** Comparison of tensile properties of BC and BC/gelatin hydrogels.

Samples	Tensile Strength (MPa)	Young’s Modulus (MPa)	Elongation at Break (%)	*p*
BC	0.600 ± 0.002	11.8 ± 0.6	6.3 ± 0.3	—
BC/gelatin	0.543 ± 0.003	10.4 ± 0.2	5.6 ± 0.3	0.05
